# Aging Well for Indigenous Peoples: A Scoping Review

**DOI:** 10.3389/fpubh.2022.780898

**Published:** 2022-02-10

**Authors:** Rachel Quigley, Sarah G. Russell, Sarah Larkins, Sean Taylor, Betty Sagigi, Edward Strivens, Michelle Redman-MacLaren

**Affiliations:** ^1^James Cook University, College of Medicine and Dentistry, Cairns, QLD, Australia; ^2^Queensland Health, Brisbane, QLD, Australia; ^3^Northern Territory Health, Darwin, NT, Australia

**Keywords:** Indigenous, aging, Indigenous health, Indigenous wellbeing, Indigenous older adults, scoping review

## Abstract

As life expectancy increases for Indigenous populations, so does the number of older adults with complex, chronic health conditions and age-related geriatric syndromes. Many of these conditions are associated with modifiable lifestyle factors that, if addressed, may improve the health and wellbeing of Indigenous peoples as they age. If models of healthy aging are to be promoted within health services, a clearer understanding of what aging well means for Indigenous peoples is needed. Indigenous peoples hold a holistic worldview of health and aging that likely differs from Western models. The aims of this review were to: investigate the literature that exists and where the gaps are, on aging well for Indigenous peoples; assess the quality of the existing literature on Indigenous aging; identify the domains of aging well for Indigenous peoples; and identify the enablers and barriers to aging well for Indigenous peoples. A systematic search of online databases, book chapters, gray literature, and websites identified 32 eligible publications on Indigenous aging. Reflexive thematic analysis identified four major themes on aging well: (1) achieving holistic health and wellbeing; (2) maintaining connections; (3) revealing resilience, humor, and a positive attitude; and (4) facing the challenges. Findings revealed that aging well is a holistic concept enabled by spiritual, physical, and mental wellbeing and where reliance on connections to person, place, and culture is central. Participants who demonstrated aging well took personal responsibility, adapted to change, took a positive attitude to life, and showed resilience. Conversely, barriers to aging well arose from the social determinants of health such as lack of access to housing, transport, and adequate nutrition. Furthermore, the impacts of colonization such as loss of language and culture and ongoing grief and trauma all challenged the ability to age well. Knowing what aging well means for Indigenous communities can facilitate health services to provide culturally appropriate and effective care.

## Introduction

The population worldwide is aging dramatically, with estimates that by 2050 the number of people aged 80 years and over will be more than 426 million, triple that of population numbers in 2020 (1). Moreover, for the first time in history, most people can expect to live into their sixties and beyond (2, 3). Increased longevity can potentially enable older adults to remain engaged for longer, resulting in positive outcomes for the individual, their families, and society as a whole. However, increasing longevity is not always associated with extended periods of good health. Epidemiological evidence indicates global health outcomes are not improving equitably, and quality of life during these extra years is unclear (2, 3). For those living with functional decline and disability, there is an increased demand on health and social care, and limitations on contributions they can make to society (3). Increased prevalence of geriatric syndromes, related to frailty, cognitive impairment, incontinence, delirium, and falls, compromise independence and increase demand for health and aged care services (4).

For Indigenous peoples, there is an increased prevalence of chronic conditions such as diabetes, cardiovascular disease, and respiratory disease (5–8). Furthermore, age-related conditions such as dementia are more prevalent in Indigenous populations and affect people at a younger age (9–12). The number of Indigenous peoples is growing (13, 14) and for many Indigenous populations, life expectancy is increasing (15). Greater proportions of Indigenous peoples over 65 years are surviving into older age (16–20). As these populations age, there may be a higher number of older adults with complex, chronic health conditions and geriatric syndromes such as falls and frailty. However, many of the problems of aging and chronic conditions are associated with lifestyle factors and are amenable to interventions that have the potential to improve the health and wellbeing of Indigenous peoples as they age.

Health is a holistic concept within Indigenous communities (21, 22). The connection to land is central to wellbeing and spiritual, environmental, ideological, political, social, economic, mental, and physical factors all play interrelated roles in wellbeing (23). When any of these factors are disrupted, ill health is likely to occur (23). Indigenous peoples therefore hold a different worldview where the concept of self is collectivist and inseparable from land, family, and community, and this view is likely to be significant in their concept of aging well.

### Concepts of Aging Well

The concept of aging well has been given considerable attention in the literature. “Aging well” is often defined synonymously with “successful ageing,” “positive ageing,” “good old age,” “active ageing,” “robust ageing,” “healthy ageing,” “productive ageing,” “vital ageing,” and “optimal ageing,” with definitions and measurements of these varying significantly (24–35). Rowe and Kahn (36, 37) are frequently cited for introducing the concept of “successful ageing,” and their model is one of the most widely used in the scientific literature (26, 27, 30, 31, 34, 38, 39). Despite its widespread use, the term “successful ageing” has generated criticism. “Success” is often associated with economic or material achievement, especially in a Western culture where the ideal of success is reflected in the lifestyles of the fortunate elite. Those who have been able to make made positive life decisions, such as financial planning and adherence to health promotion advice, are rewarded, and those who find themselves in less favorable circumstances often experience blame or neglect. The concept of success or failure to categorize aging promotes the notion of winners and losers, rather than successful aging being on a continuum of achievement (24, 28, 29, 32, 39, 40).

The model of successful aging posited by Rowe and Khan (36) is based on three criteria: (i) freedom from disease and disability; (ii) high cognitive and physical functioning; and (iii) active engagement with life. These criteria place an emphasis on maintaining physical health and avoiding disease. However, this approach has been criticized for being a medically-orientated model which: (i) neglects social relationships and engagements (41–43); (ii) underestimates the influence of contextual factors (35, 41, 44, 45); (iii) is oblivious to cultural differences (35, 40, 41, 43, 46); (iv) ignores the interactions between individuals and their environments (43–45); (v) overlooks mental wellbeing (38, 43); (vi) fails to take into account the disadvantages that accumulate over the life course (25, 29, 40, 43–45); (vii) fails to consider the developmental process throughout the individual's lifespan (25, 29, 40, 43, 46); and (viii) does not acknowledge that successful aging is possible for those with chronic disease or disabilities (29, 38, 41–43, 45). Incorporating broader, non-medical perspectives in models of aging well can enhance theoretical definitions and enable more individual- and community-centered definitions of aging well to emerge. Studies that have encompassed lay perspectives found non-medical models of aging well to be multidimensional, with a greater focus on adaptation, meaningfulness, and connection, and provide insight into what older people value (25, 26, 29, 30, 33, 41, 42, 47). Knowing how aging well is expressed within particular communities allows health and social care providers to provide the most appropriate health care (33). It is therefore imperative that aging well is considered from a cultural perspective.

Dimensions of aging well for different cultural groups have been explored across the literature. Prior research overwhelmingly reports that older people's norms, perceptions, and self-awareness of the reality of aging differ across cultures, making aging well culture-dependent (28, 29, 44, 48). Hung and colleagues (28) compared the concept of healthy aging from Western and non-Western cultural perspectives, as well as between academics and lay older persons. They found older people from non-Western cultures held a more holistic view of healthy aging, which extended beyond functional independence, and included domains such as family, adaptation to age-related changes, financial security, personal growth, positive spirituality, and positive outlook. The authors suggested attitudes and behaviors relevant for healthy aging are greatly influenced by traditions, religious beliefs, and values derived from different individual cultural backgrounds, yet current academic definitions of healthy aging seem to be independent of cultural identity. Amin (41) explored successful aging from older adults' perspectives in Bangladesh and found, similarly to Hung and colleagues (28), that successful aging encompassed dimensions such as adaptations to one's changing body, financial security, religiosity, age identity, and social engagement. Amin (41) found that older adults' emphasis on these dimensions, however, was qualitatively different from those identified as relevant in Western societies, and that family relationships played a strong role in aging success—something often neglected in Western models.

Although some domains of aging well, such as physical health and economic wealth, may be consistent across cultures, their relative contributions to wellbeing vary. Other more culturally situated values, such as transferring cultural knowledge or participating in cultural activities, may hold greater importance in certain cultures (29, 34). Incorporating perspectives from different cultural settings will facilitate construction of a more comprehensive, culturally appropriate definition of aging well, which would be more realistic and useful for the community in which it is developed (28). Additionally, an understanding of aging well may support both health and social care systems to be better aligned to integrate services that support a holistic view of functioning and healthy aging (3). If models of healthy aging are to be promoted within health and social care services, there needs to be a clearer understanding of what aging well means for Indigenous peoples. This is particularly significant for Indigenous people where healthy aging may not be easily achieved (7, 49).

### Purpose of Review

This review was conducted as part of the lead author's PhD study to develop and implement a framework for aging well in the Torres Strait in Far North Queensland, Australia. The islands of the Torres Strait are located between the northern tip of Australia and Papua New Guinea. There are over 100 islands with Torres Strait Islanders permanently living in 18 island communities and two mainland communities on the Northern Peninsula Area (the northern most tip of Australia) (50). Torres Strait Islander peoples are a culturally distinct First Nation population in Australia, predominately of Melanesian ethnicity but due to the settlement of traders, explorers, and colonizers have a diverse and mixed ancestry (50). Torres Strait Islander peoples are sea-faring people whose culture has been influenced by peoples from Australia, Papua, and the Austronesian region (51). Early Torres Strait life was based on subsistence living with communal life revolving around hunting, fishing, gardening, and trading. Fishing remains the main economic activity across the region (50). The PhD study, to collaborate with local primary health care centers in the Torres Strait region to develop an aging well framework, follows a longstanding research and clinical partnership with local health services and community groups. Community members expressed a desire to examine what aging well meant for their older adults and how they could be supported to age well into the future. The findings of this review were used to assist with analysis of local needs and priorities.

### Scope and Aims of Review

Globally, there are between 370 and 500 million Indigenous peoples who live in over 90 countries (8). Within specific Indigenous populations, there are often different cultural groups, each with distinct culture, language, beliefs, and practices. This scoping review included articles relating to many Indigenous populations, each with preferred terminology when referring to their people. The lead author of the review is a non-Indigenous clinical researcher supported by a wider research team and advisory panel of Indigenous and non-Indigenous academics, researchers, and clinicians. Together, the author group respectfully agreed that using the term Indigenous throughout the review would be inclusive to all participants across all studies.

Aspects of aging well relating to specific Indigenous groups have been documented, and a scoping review has been conducted on exploring successful aging amongst North American older Indigenous peoples (52). However, no systematic review has been conducted to explore similarities across wider Indigenous populations. The intent of this paper is not to suggest all Indigenous peoples hold the same perspectives of aging well and that a generic Indigenous model of aging well can be developed. The aims of this scoping review were to: (i) explore what aging well means for different Indigenous populations; (ii) compare concepts of aging well for these populations with non-Indigenous perceptions; and (iii) identify gaps in the literature on aging well for Torres Strait Islander populations to inform further research.

The following questions were developed to meet the aims of the review:

1. What literature exists and where are the gaps on what aging well means to Indigenous peoples?
2. What is the quality of the literature on aging well for Indigenous peoples?
3. What are the domains of aging well for Indigenous peoples?
4. What are the enablers and barriers to aging well for Indigenous peoples?

## Methods

A scoping review methodology was selected as the preferred approach as it allows broad concepts to be addressed, generates key concepts, and identifies gaps in the literature whilst using a structured systematic methodology (53, 54). Furthermore, scoping review methodology enables the inclusion of both qualitative, quantitative, and mixed methods studies (53, 54). The scoping review methodology outlined by Arksey and O'Malley (53) and enhanced by Levac et al. (55), was employed. This methodology increases the rigor and reliability of review findings (53). As recommended by Levac et al. (55) and Daudt et al. (56), a quality assessment component was included in the review. The Quality Assessment Tool for Studies with Diverse Designs (QATSDD) (57) was applied to assess the quality of included literature. However, as scoping reviews are intended to capture a broad range of literature regardless of study design (53, 54), no studies were excluded from this review based on the quality appraisal. The PRISMA Extension for Scoping Reviews (PRISMA-ScR) guidelines and checklist were used to guide the reporting for this scoping review (58).

### Literature Search

The search strategy included an electronic database search and a website search. Search terms from key relevant publications were reviewed to identify key words in the areas of aging and Indigenous populations. The search was broadened or focused using truncation symbols and Boolean connectors AND, OR, NOT. The following databases were searched using MeSH headings or key words: Medline, CINAHL, PsycInfo, Emcare, PubMed, Embase, Scopus, and Informit. Reference lists of articles identified through searching of databases were reviewed to identify possible additional sources. A search for gray literature was conducted on the World Wide Web, on websites such as the World Health Organization and on Indigenous-specific websites, such as the Australian Institute of Aboriginal and Torres Strait Islander Studies, Health Info Net, and the Lowitja Institute.

### Study Selection

Publications identified in the search were included if they focused on perceptions, attitudes, or experiences of aging, or domains of aging well, or barriers and enablers of aging well for Indigenous peoples. Aging well and all associated terms were included; successful aging, positive aging, good old age, active aging, robust aging, healthy aging, productive aging, vital aging, optimal aging, and harmonious aging. The United Nations states that there is no formal universal definition of “Indigenous peoples” but Indigenous peoples are identified by their social, cultural, economic and political characteristics that are distinct from those of the dominant societies in which they live and are frequently marginalized within their own countries (8, 59). Articles were included where participants were described as Indigenous or recognized other titles for example First Nations, Aboriginal, Māori, Alaska Natives, and American Indians.

Publications identified in the searches were charted in Microsoft Excel for evaluation and eligibility assessment (54). The lead author (RQ) screened titles and abstracts using the inclusion/ exclusion criteria outlined in Table 1. Two authors (RQ, SR) independently read the full text of articles, applying the inclusion/exclusion criteria, and described the study characteristics. Where outcomes of eligibility assessment differed and remained unresolved after discussion, authors MRM or SL were consulted for a consensus agreement. Publication characteristics charted were source of article, year of publication, Indigenous population of study participants, aim or purpose of study, methodology including design and analysis of data, and study outcomes.

**Table 1:** Inclusion/exclusion criteria for scoping review papers.

**Criterion**	**Inclusion**	**Exclusion**
Publication focus	Perceptions of, attitudes to, concepts of, cultural aspects of, definitions of, aging well and associated terms. Discussion of domains of aging well. Barriers and enablers to aging well.	Perspectives of aging well (and associated concepts) or measures of aging well of Indigenous peoples that were incorporated into wider cultural groups. Focus on specific diseases of aging. Focus on cellular or biological aging. Focus on older age but not perspectives of aging well (or associated terms).
Population	Indigenous peoples worldwide	
Language	Published in English	
Time period	Published between 2000 and 2020	
Type of article	Original research including qualitative, quantitative and mixed methods. Gray literature, government, peak bodies or organizational reports, website information. Full text available.	Literature reviews (relevant articles from these included), commentaries, editorials, book reviews, letters to the editor, or where the full text was not available.

### Quality Appraisal

The aim of critical appraisal within a systematic review methodology is to evaluate whether the studies included in the review are accurately and completely described to assess for validity, rigor, and trustworthiness (60). Including a quality appraisal of publications within a scoping review methodology can assist with interpretation of results and facilitate the uptake of findings into policy and practice (55, 56, 61). The QATSDD is a 16-item tool that generates scores from 0 to 42 and has demonstrated good reliability and validity for use in the quality assessment of a diversity of studies, which include qualitative and quantitative work (57). Two authors, RQ and SR independently applied the tool to the 32 included studies. Where discrepancies in scores arose, consensus was reached through discussion. The scoring outcomes are included in Table 2.

**Table 2:** Characteristics of included publications (*N* = 32).

**References**	**Title**	**Indigenous population**	**Aims**	**Study design and methodology**	**Summary of findings**	**QATSDD score**
Abonyi and Favel (62)	Marie's story of aging well: Toward new perspectives on the experience of aging for Aboriginal seniors in Canada.	Metis, Canada	To consider the construction of a framework of healthy aging for Aboriginal peoples in Canada.	Conference paper Biographical account	Documented the significance of ongoing contributions to community life, transmission of accumulated knowledge, and wisdom to younger generations and the connection with cultural traditions.	N/A
Baron et al. (63)	Aging, health and place from the perspective of Elders in an Inuit community.	Inuit, Canada	To explore the perspectives of Inuit Elders on the relationship between aging, health and place.	Qualitative In-depth interviews with Inuit Elders aged 50–86 (*n* = 20) Thematic analysis	Documented spending time with children, having social support, living in houses adapted to aging health conditions, having access to community activities and services, and time spent on the land as the main resources supporting health. Stressed the importance of being able to grow old in their own community.	22
Baron et al. (64)	The social determinants of healthy aging in the Canadian Arctic.	Inuit, Canada	To identify social determinants of health associated with healthy aging.	Quantitative Survey data from a larger national survey Respondents aged over 50 (*n* = 850) Holistic indicator of healthy aging used Descriptive analyses used including multivariate multinomial regressions	Social determinants of health associated with the “Good health” profile related more to social relationships and participation, those associated with the “Intermediate health” profile related more to economic and material conditions.	32
Baskin and Davey (65)	Grannies, elders, and friends: Aging Aboriginal women in Toronto.	First Nations, Inuit and Metis, Canada	To further the knowledge about seniors/Elders on their roles; perspectives on aging, health, and wellbeing; concerns; and needed services.	Qualitative Story-telling circle (*n* = 10) and individual interviews (*n* = 2) with women aged 60–75 Thematic analysis	Documented the use of humor and laughter, ongoing processes of teaching and learning, effects of residential school system, value of kinship and community relationships, and friendships.	19
Boyd (66)	“We did listen.” Successful aging from the perspective of Alaska Native Elders in Northwest Alaska.	Alaska Natives, United States of America	To establish a deeper understanding of how Alaska Native Elders in Northwest Alaska understand and experience successful aging to inform program development and service delivery.	Qualitative (Thesis) Phenomenological study Semi-structured interviews with Elders (*n* = 14)	Documented engagement with family and community, self-awareness and care, and a sense of gratitude as essential elements of successful aging. Elders who age successfully listened to and learned from their Elders, enact traditional values and practices, and pass wisdom and knowledge to future generations.	42
Brooks-Cleator and Lewis (67)	Alaska Native Elders' Perspectives on physical activity and successful aging.	Alaska Natives, United States of America	To explore how Alaska Native Elders perceive the role of physical activity as they age and its contribution to successful aging.	Qualitative Semi-structured interviews (*n* = 41) Thematic analysis	Documented being physically active is important for successful aging. Being an Elder means being able to actively participate in subsistence activities and teach others subsistence. Engaging in physical activity was not just seen as a personal responsibility to maintain health and age successfully, but also to improve or maintain physical, mental, emotional, and spiritual health; and/or to enable continued participation in subsistence activities rooted in their culture and traditional roles as Elders.	38
Brooks-Cleator et al. (68)	Community-level factors that contribute to First Nations and Inuit older adults feeling supported to age well in a Canadian city.	First Nations and Inuit, Canada	To address what community-level factors contribute to Indigenous older adults (aged 55 years and over) feeling supported to age well in the city of Ottawa.	Qualitative CBPR^*^ approach Semi-structured interviews, focus groups, and photovoice with First Nations and Inuit older adults (*n* = 32) Thematic analysis	Documented two main areas in which participants felt they could be better supported to age well: the social environment (responsive health and community support services, respect and recognition, and communication and information) and physical environment (transportation, housing, accessibility, and gathering space).	37
Browne et al. (69)	Listening to the voices of native Hawaiian Elders and ‘Ohana caregivers: Discussions on aging, health, and care preferences.	Native Hawaiian, United States of America	To investigate health and care preferences that offer the potential for improving wellbeing in later life for Native Hawaiian Elders.	Qualitative Semi-structured listening meetings (*n* = 6), involving community-dwelling kupuna (*n* = 24) and ‘ohana caregivers (*n* = 17) aged 60–94 Constant comparative method of analysis	Documented challenges with aging and caregiving and the influence of culture and social stressors on health needs and care preferences. Affordable, accessible, and acceptable programs and policies that can respond to the growing health and care needs of native elders and family caregivers are needed.	35
Browne and Braun (70)	Away from the islands: Diaspora's effects on Native Hawaiian Elders and families in California.	Native Hawaiian, United States of America	To examine reasons for migration and perspectives on aging and caregiving in a sample of Native Hawaiian Elders and family caregivers residing in Southern California.	Qualitative Key informant interviews (*n* = 10) and kupuna and ‘ohana caregivers focus group (*n* = 20) Constant comparative method of analysis	Documented concerns about challenges associated with aging and caregiving, and how cultural traditions and values continue to shape caregiving and service preferences.	38
Butcher and Breheny (71)	Dependence on place: A source of autonomy in later life for older Maori.	Māori, New Zealand	To examine the ways that place influences experiences of aging for older Māori in New Zealand.	Qualitative Interviews with participants aged 66–79 (*n* = 8) Thematic analysis	Documented attachment to place provided the foundation for experiences of aging. Through connection to place, the participants drew on a comforting and comfortable dependence on land and family to enable autonomy in later life. A good old age depended on balancing competing demands of living in wider society with attachment to place and Māori identity in later life.	24
Collings (72)	“If you got everything, it's good enough:” Perspectives on successful aging in a Canadian Inuit community.	Inuit, Canada	To examine Inuit definitions of successful and unsuccessful aging.	Qualitative Structured interviews (*n* = 38)	Documented successful old age is not characterized by individual good health, but by the ability to successfully manage declining health. Important determinants of a successful Elderhood are not material but ideological, such as, attitudes in late life, willingness to transmit wisdom and knowledge to juniors.	20
Coombes et al. (49)	First Nation Elders' perspectives on healthy aging in NSW^**^, Australia.	Aboriginal and Torres Strait Islander, Australia	To examine the perspectives of Australian First Nation people about healthy aging.	Qualitative Yarning Circles (*n* = 8) with adults aged 45 and over (*n* = 76)	Documented key issues around healthy aging including; the impact of chronic disease, community and connections, sharing knowledge of history and culture. Barriers to aging well-described. Healthy aging viewed as the ability to continue in key roles as cultural leaders and the keepers of traditional knowledge.	32
Edwards (73)	Taupaenui Māori Positive Aging.	Māori, New Zealand	To explore the characteristics of positive Māori aging.	Qualitative (Thesis) Semi-structured interviews with older Māori (*n* = 20) Thematic analysis	Documented Māori-specific domains of successful aging are stewardship, connectedness transmission, contribution, adaptability, and self-determination with the overarching theme of realized potential.	36
Gallardo-Peralta and Sanchez-Moreno (74)	Successful aging in older persons belonging to the Aymara native community: Exploring the protective role of psychosocial resources.	Aymara, Chile	To analyse the process of successful aging in older persons.	Quantitative Cross-sectional Questionnaire to Aymara aged >60 (*n* = 232) comprised of validated measurement scales for successful aging. Descriptive statistics and a hierarchical regression analysis for the successful aging	Documented successful aging is positively related with community integration, social support from informal systems (social groups), quality of life, and religiousness (forgiveness). In contrast, successful aging is negatively related with depression.	39
Hopkins et al. (75)	Keeping busy: a Yup'ik/Cup'ik perspective on health and aging.	Alaska Natives, United States of America	To explore cultural beliefs and practices of health and wellbeing of Yup'ik/Cup'ik women in two rural villages in southwestern Alaska.	Qualitative Semi-structured interviews with females aged 38–89 (*n* = 15) Thematic analysis	Documented healthy aging is defined within the framework of subsistence living; keeping busy, walking, eating subsistence foods, and respect for elders. These beliefs and practices promote a strong, active body and mind as vital components to healthy aging.	23
Laditka et al. (76)	Attitudes about aging well among a diverse group of older Americans: Implications for promoting cognitive health.	American Indian, United States of America	To examine perceptions about aging well in the context of cognitive health among a large and diverse group of older adults.	Qualitative 42 focus groups with 4 American Indian focus groups (*n* = 34) Constant-comparison methods were used to analyze the data by ethnic group	Documented American Indians did not relate aging well to diet or physical activity. Aging well-included; living to advanced age, having good physical health, having a positive mental outlook, being cognitively alert, having a good memory, and being socially involved.	21
Lewis (77)	Successful aging through the eyes of Alaska Natives: exploring generational differences among Alaska Natives.	Alaska Natives, United States of America	To explore the concept of successful aging from an Alaska Native perspective, or what it means to age well in Alaska Native communities.	Qualitative Interviews with participants aged 26–84 from 6 tribal communities (*n* = 15) Grounded theory	Documented aging successfully is based on local understandings about personal responsibility and making the conscious decision to live a clean and healthy life. Poor aging characterized by a lack of personal responsibility, or not being active, not being able to handle alcohol, and giving up on oneself.	20
Lewis (78)	Successful aging through the eyes of Alaska Native Elders. What it means to be an Elder in Bristol Bay, AK^***^.	Alaska Natives, United States of America	To explore successful aging from an Alaska Native perspective or what it means to reach “Eldership” in rural Alaskan communities.	Qualitative Interviews with participants aged 61–93 (*n* = 26) Thematic analysis	Documented four elements of “Eldership” or what Alaska Native Elders believe are important characteristics to becoming a respected elder; emotional wellbeing, community engagement, spirituality, and physical health.	30
Lewis (79)	The importance of optimism in maintaining healthy aging in rural Alaska.	Alaska Natives, United States of America	To develop a model of successful aging for Alaska Native Elders in Bristol Bay, Alaska.	Qualitative Interviews with participants aged 61–93 all Elders from 6 communities (*n* = 26) Grounded theory	Documented four themes of successful aging: emotional wellbeing, community engagement, spirituality, and physical health A positive outlook on life was found in each of the four elements of successful aging.	24
Lewis (80)	The future of successful aging in Alaska.	Alaska Natives, United States of America	To explore the concept of successful aging from a younger urban Alaska Native perspective and explore if they believe they will achieve a healthy older age.	Qualitative Interviews with participants under 50 years from 4 Alaskan Native tribal groups (*n* = 7) Grounded theory	Documented Alaska Natives see the inability to age well as primarily due to the decrease in physical activity, lack of availability of subsistence foods and activities, and the difficulty of living a balanced life in urban setting.	22
Lewis (81)	The role of the social engagement in the definition of successful aging among Alaska Native Elders in Bristol Bay, Alaska.	Alaska Natives, United States of America	To explore the role of social engagement (family and community support) in Alaska Native Elders' definitions of successful aging, why social engagement is important to the health and wellbeing of Alaska Native Elders.	Qualitative Interviews with Elders (*n* = 25) Content analysis	Documented the importance of family and community, not only as a source of support but also as part of their culture and identity. Providing family support sustained meaningful roles, which contributed to wellbeing, optimism and generative behaviors.	33
Lewis (82)	What Successful Aging Means to Alaska Natives: Exploring the reciprocal relationship between the health and wellbeing of Alaska Native Elders.	Alaska Natives, United States of America	To highlight the role of the community in Alaska Native Elders' definitions of successful aging, and explores how the Elders contribute to the health and resilience of rural communities.	Qualitative Interviews with 26 Elders (*n* = 26) Grounded theory	Documented the importance of family and community support, which contributes to optimistic attitude toward life. This support provides the Elders with a sense of purpose and having a role in their family and community, directly impacting their health and wellbeing, and enabling them to remain active in their homes and communities.	32
Pace (83)	Meanings of memory: Understanding aging and dementia in First Nations communities on Manitoulin Island, Ontario.	First Nations, Canada	To understand expectations for successful aging among Aboriginal peoples on Manitoulin Island.	Qualitative CBPAR^*^, Ethnography and Phenomenology Semi-structured interviews with seniors, people with dementia, informal family caregivers, health care providers, and traditional healers in seven First Nations communities. Focus groups with nurses and personal support workers	Documented aging as a natural process. A successful old age characterized by: acceptance, good overall health, making an effort to maintain health through behaviors such as exercise, eating well and avoiding alcohol and tobacco, staying engaged in social activities, participating in spiritual and cultural activities, having a positive attitude and a sense of purpose, and maintaining autonomy.	40
Pace (84)	“Place-ing” dementia prevention and care in NunatuKavut, Labrador.	Inuit, Canada	To explore experiences of transitions into aging and dementia in NunatuKavut, Labrador.	Qualitative CPAR^*^ Photovoice approach using interviews with participants aged 50 > (*n* = 14) Phenomenological thematic analysis	Documented the prominence of culture and the natural environment in descriptions of health promotion and care trajectories. These factors may contribute to healthy aging, protect against cognitive decline, and support the maintenance of identity for people living with dementia.	33
Pearse et al. (85)	Growing old in Kempsey: Aboriginal people talk about their aging needs.	Aboriginal, Australia	To seek more information about the aging needs of Aboriginal people on the North Coast of New South Wales, Australia and explore the lived experience of Aboriginal people in Kempsey as they age.	Qualitative (Report) Semi-structured interviews with participants from 9 communities (*n* = 30) Thematic analysis	Documented that family relationships and culture are important. Raising grandchildren is valuable. Barriers faced in later age also documented.	25
Radford et al. (15)	Sharing the wisdom of our Elders; Final report.	Aboriginal and Torres Strait Islander, Australia	To highlight the healthy aging stories from the participants of the Koori Growing Old Well Study (KGOWS)	Qualitative (Report) Integrated quantitative findings from the KGOWS cohort 118 responses to an open ended survey question and semi-structured interviews with service providers	Documented themes to aging well that included: Connections to Country and culture; respect yourself, the Elders and all the mob; resilience; getting together, yarning, passing on knowledge; keeping healthy to live a long life; saying no to smoking, alcohol and drugs; and education.	24
Ranzijn (86)	Active aging-another way to oppress marginalized and disadvantaged elders? Aboriginal Elders as a case study.	Aboriginal, Australia	To question whether the concept of active aging unintentionally devalues the life experiences of disadvantaged groups of older people.	Qualitative 5 yarning circles with participants (*n* = 20)	Documented that active aging presents a narrow image of aging, which does not accord with the experiences and priorities of many older people, and it alienates large groups of marginalized older people and reinforces social exclusion. A model of aging, around the concept of “authentic ageing,” that respects and acknowledges the unique and valued role of elders which encompasses more than aging bodies is preferable.	10
Smith et al. (87)	Inupiaq Elders study: aspects of aging among male and female elders.	Alaska Natives, United States of America	To determine if age and gender subsets of Elders in urban and rural locations present differences in self-reported health, physical and mental functioning, functioning of daily activities, body mass index, nutrient intake and food insecurity.	Quantitative Comparative survey of Inupiaq Elders (*n* = 100)	No significant differences were found by age, gender or location for demographic variables. Data indicate that Alaskan Inupiaq Elders are aging well and reporting few physical and mental problems.	37
Waters and Gallegos (88)	Aging, health, and identity in Ecuador's Indigenous communities.	Indigenous, Ecuador	To investigate the perceptions regarding the ability of family and community networks to provide adequate and appropriate support for older persons in the context of their perceptions of health, health care, and aging.	Qualitative Focus groups (*n* = 15) with participants aged 60> (*n* = 148) interviews with community leaders, local health care professionals, and traditional healers (*n* = 10) Grounded theory	Documented that aging defined as successful in terms of capacity to work the land and participate actively in community affairs. Perceptions of aging are shaped by chronic illness, fatigue, deteriorating sensory capacities, and vulnerability to accidents. Barriers to health care are exacerbated among aging members of indigenous communities.	28
Waugh and Mackenzie (89)	Aging well from an urban Indigenous Australian perspective.	Aboriginal and Torres Strait Islander, Australia	To explore perspectives of older Indigenous Australians about their health and wellbeing.	Qualitative Interviews with participants aged over 45 yrs (*n* = 6) Phenomenology	Documented important considerations for aging well that related to four main themes of: personal identity, family, community, and perception of health and aging.	36
Wettasinghe et al. (90)	Older Aboriginal Australians' health concerns and preferences for healthy aging programs.	Aboriginal and Torres Strait Islander, Australia	To explore participants' health concerns, preferences for healthy aging programs, and receptiveness to technology.	Qualitative Semi-structured interviews with Aboriginal and Torres Strait Islanders Australians aged >50 years from regional and urban communities (*n* = 34) Grounded theory approach.	Documented that a successful healthy aging program model includes physical and cognitive activities, social interaction, and health education. The program model also provides culturally safe care and transport for access as well as family, community, cultural identity, and empowerment regarding aging well as central tenets.	35
Wright-St. Clair et al. (43)	Ethnic and gender differences in Preferred Activities among Māori and non- Māori of advanced age in New Zealand.	Māori New Zealand	To explore active aging for self-nominated important everyday activities.	Quantitative Participants in the LiLACS^****^ study (*n* = 649, 252 Māori and 397 non-Māori) Activities were coded and categorized and then put into one of nine domains.	Important activities for older Māori people were: gardening, reading, walking, cleaning the home, organized religious activities, sports, extended family relationships, and watching television.	23

* *Community-Based Participatory Action Research*.

** *New South Wales*.

*** *Alaska*.

**** *Life and Living in Advanced Age Cohort Study*.

### Analysis

A coding framework was developed by the authors based on the literature and research questions. Data from all extracted papers were deductively coded using the framework, employing NVivo 12 qualitative data software V12 (QSR International) to manage the data. Themes were created based on the identification of patterns from the coded data through use of reflexive thematic analysis methodology (91, 92). Themes and potential domains of aging well were discussed between RQ, MRM, and SR until consensus was achieved and the final themes derived.

## Results

### Search Results

**Figure 1:**
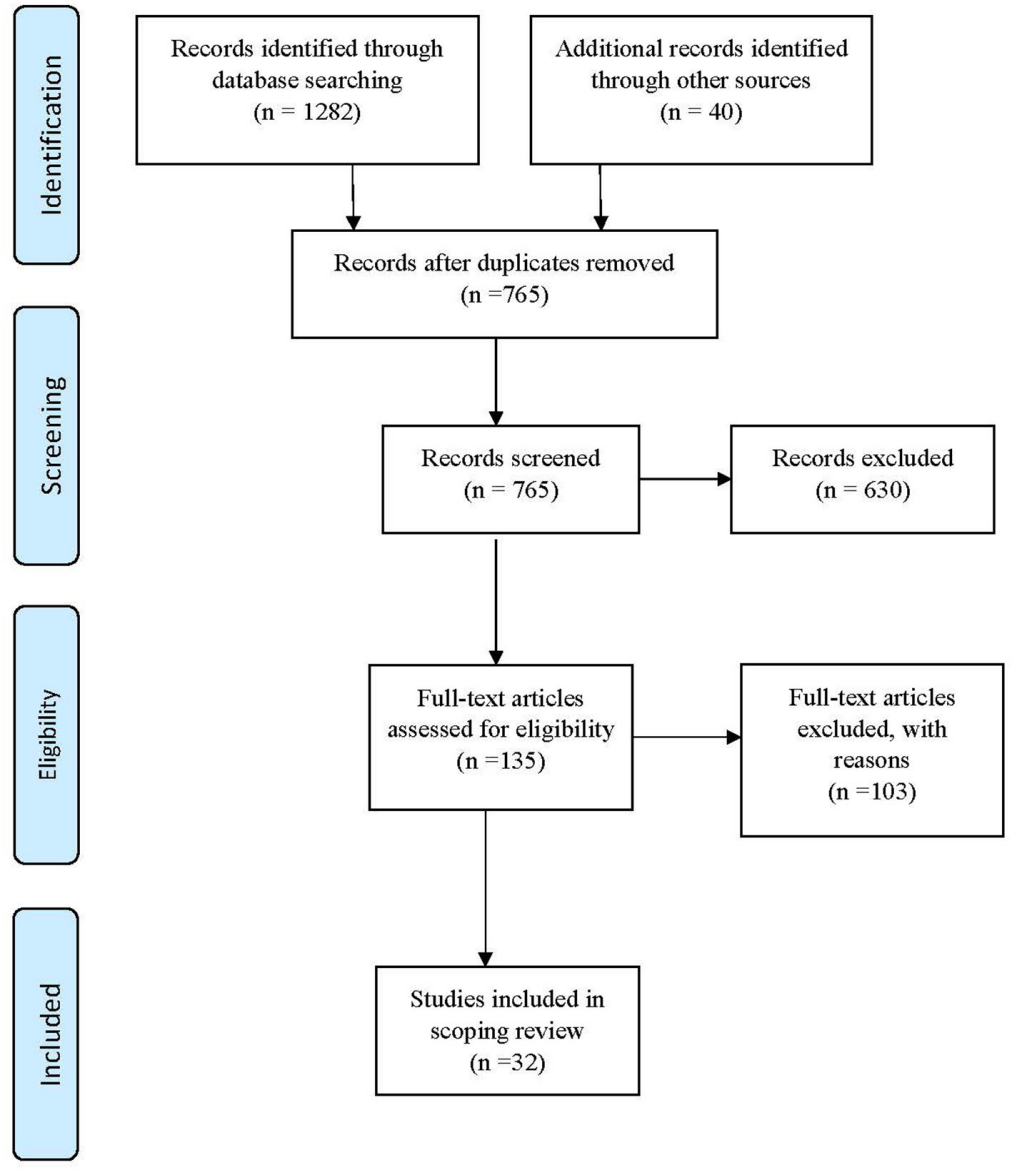
PRISMA flow chart (93).

Database searching identified 1,282 potential papers with 40 articles identified through additional sources. After the exclusion of duplicates, 765 were subjected to title and abstract review. Of these, 135 publications were selected for full text review with 32 of these publications meeting the inclusion criteria and included in the review (Figure 1).

### Description of Studies

Table 2 provides a summary of the characteristics of the 32 included publications. The majority of the publications (*n* = 27) used qualitative methodology, four publications used quantitative methodology, and one publication was a biographical account. Twenty-seven articles were published in peer reviewed journals, two were published reports, and three were published theses. Indigenous populations that were the focus of the included studies were: Metis, Canada (*n* = 2); Inuit, Canada (*n* = 6); First Nations, Canada (*n* = 3); Alaska Natives, United States of America (USA) (*n* = 10); Native Hawaiian, USA (*n* = 2); Aboriginal and Torres Strait Islander, Australia (*n* = 6); Maori, New Zealand (*n* = 3); Aymara, Chile (*n* = 1); American Indian, USA (*n* = 1); and Indigenous, Ecuador (*n* = 1).

### Quality of the Included Studies

The methodological quality of the included publications was assessed from 10 through to 42 (average = 29). Whilst the biographical account (62) did not fit into a methodological framework and was therefore rated N/A, it was important to include it to ensure all Indigenous voices were heard. The three published theses (66, 73, 83) all scored highly as they included detailed methodological procedures. A common limitation of the qualitative studies was a lack of evidence to determine reliability of the analytical process (15, 49, 62, 63, 65, 75–77, 79, 80, 84–86). Other limitations noted across all study designs included lack of evidence of co-design (62, 64, 65, 72, 76, 80, 86, 88, 94), evidence of sample size considered in terms of analysis (62–65, 67–69, 71, 72, 75, 77, 81, 82, 84–86, 90, 94) and detailed recruitment data (62, 63, 69, 71, 72, 76–81, 85, 86, 94).

## Synthesis of Findings

**Figure 2:**
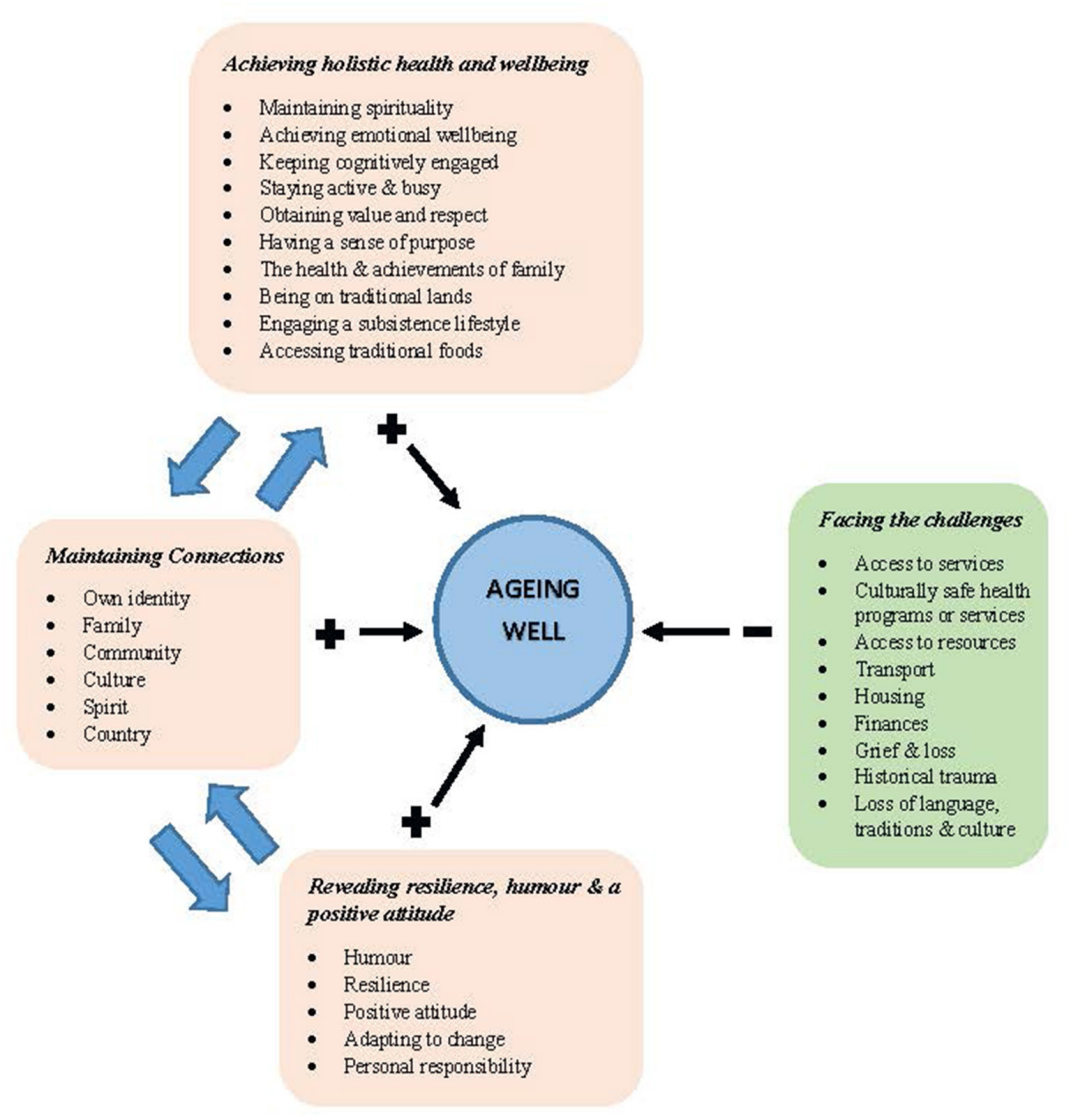
Concept of ageing well as described in the scoping review.

Across all Indigenous populations, there were consistently shared similarities that reflected perceptions of what aging well means for Indigenous peoples and challenges that impacted Indigenous peoples' ability to achieve good health and wellbeing in later life. Four major themes were identified: (1) achieving holistic health and wellbeing; (2) maintaining connections; (3) revealing resilience, humor, and a positive attitude; and (4) facing the challenges. These themes are interrelated, each having influence on, and being influenced by, the other themes, demonstrating that aging well is a holistic concept with reliance on connections to person, place and culture and influenced by the social determinants of health (Figure 2).

### Achieving Holistic Health and Wellbeing

To achieve health and wellbeing in later life, several factors were identified that contributed to the perception of aging well. Those factors included: maintaining spirituality (15, 66, 69, 73, 74, 77–79, 83); achieving emotional wellbeing (15, 72, 73, 75, 77, 79, 80); keeping cognitively engaged (66, 73, 76, 83–85); staying active and busy (15, 49, 64, 66, 67, 73–76, 83, 87); obtaining value and respect (15, 66, 68, 73, 75, 77, 78, 89, 90); and having a sense of purpose (83, 88, 90, 94). Furthermore, the impact of the health and achievements of the wider family (71, 73, 85), being on traditional lands (15, 63, 71, 73, 80, 81, 84, 87), engaging in subsistence lifestyles (66, 67, 71, 75, 84), and accessing traditional and healthy foods (15, 66, 69, 70, 73, 75, 77, 78, 81, 83, 87) all contributed to the attainment of health and wellbeing in older age. These concepts are described in the following sub-themes of: everything healthy—mind, body and spirit; my job is done; and big stress relief when out on land. Overwhelmingly, however, it was a combination of all of these factors, along with a holistic approach to a healthy lifestyle, which contributed to holistic health and wellbeing (15, 66, 70, 72, 73, 75, 80, 83, 84, 89, 90), that is described in the sub-theme of balance—foundations for a long life.

#### Everything Healthy—Mind, Body, and Spirit

Having a good spirit was identified as providing a balance in life and a sense of strength (83). Through maintaining spirituality, there was a sense of guidance toward how to age well (15, 66, 73, 74, 79, 83) while alleviating worry (78), and keeping an optimistic (69) and positive attitude (15, 78).

“*… one of the things that's been most important in my life has been my spirit…it is something that I had taken care of because to me it's the governing body. If I hadn't got that right, I don't think anything goes right.”* [Older Maori, (73) p. 189]

Having emotional stability and being satisfied with past life decisions was viewed as an indicator of aging well (15, 72, 73). A poor mental state was reported as due to too much worry (72, 77).

“*Ones that don't worry too much stay young. Ones that worry too much age faster.”* [Alaskan Native Elder (79) p. 1,525]

Staying cognitively active was part of a holistic approach to aging well (66, 73), achieved through lifelong learning (15, 73) and engagement in cognitively stimulating activities (66, 73, 76, 83–85).

“*I'm more into puzzles, using my head or brain to figure out things”* [Older First Nations Canadian, (83) p. 68]

Physical health was viewed as important (15, 49, 66, 67, 73, 75, 83) but valued through appreciating how engagement in physical activities could provide opportunities for social connectedness (83), providing a sense of purpose (66, 73, 83, 88), improving quality of life (73, 90), promoting cultural continuity (64), and, in a practical sense, providing access to food (64). Physical activities were rarely described as formal or structured, such as visits to a gym, but were more likely to be achieved as a consequence of enacting traditional and subsistence living (64, 66, 67, 74, 75, 83), involvement in community activities (66, 76, 83, 87, 90, 94) or carrying out activities of daily living (66, 83).

“*I think today most of the women are healthy for activity, physical activities. When they go berry picking, they're working using their bodies everything. When we are cutting fish, we are using everything, our muscles, lifting things”* [Older Native Alaskan, (75) p. 45–6].

#### My Job is Done

As part of a holistic approach to aging well, obtaining recognition and respect from both family and communities was viewed as significant (15, 68, 73, 75, 77, 78, 89, 90). Having an appreciation of the wealth of knowledge and wisdom an older adult can share elicited feelings of honor and pride and made the older adults feel supported and valued in the work they do (15, 66, 73).

“*…they get to feel that they are still important, that they are of value that they have something of value to still give. You know, that they're not just pushed aside. You see I'm always conversing with my older ones, you know, that their opinion is important.”* [Older Maori, (73) p. 205]

Equally, the wellbeing and achievements of the wider family unit contributed to older adults' measures of aging well. The accomplishments of children and grandchildren created a sense of pride and satisfaction for the older adult (71, 73, 85).

“*What makes me happy is seeing the family happy, all my family. You know all of us, all the kids. Yeah, yeah that's what makes me happy. I don't really need that much for myself”* [Older Maori (71) p. 53]

#### Big Stress Relief When Out on Land

Being on traditional lands was found to promote wellbeing, which in turn influenced perceptions of aging well (15, 63, 71, 81, 84, 87). Not only a place of childhood memories (63), being on traditional lands also signified a symbolic connection to culture and language (15, 63, 73). There was a deep and fundamental connection to the natural environment that facilitated both health and mental balance and where continued engagement was central to aging well (15, 73, 80).

“*When she's on the land too that's when her stress is all come out, like less stress, good stress relief, because that's where she pretty much grew up so it's big stress relief when she finally goes out on the land.”* [Older Inuit (63) p. 140]

Several studies discussed the contribution that subsistence living made to aging well (66, 67, 71, 75, 84). A subsistence way of living was seen as a healthy way to age for several reasons including: as a way to engage with family, a means to look after the environment, opportunity to connect to the land and to connect to past generations whilst engaging in physical exercise and providing traditional food, clothing, and shelter (66, 67, 75, 84). Furthermore, participation in subsistence living gave a sense of identity and connection for the older adult to themselves and others (66).

“*every spring… we start to gather off of the land and that's what keeps our Elders healthy”* [Alaskan Native Elder, (67) p. 299]

Access to traditional foods also played a part in perceptions of aging well (66, 69, 70, 73, 77, 78, 81, 83, 87). Traditional foods were viewed as healthy, making both the mind and spirit strong. Food was more than nourishment, symbolizing a connection to culture through traditions and ceremonies (69).

“*In years back, before I was born, I know there were elders that were very healthy and strong because they have their food, their native food, not mixed up with the kass'aq [white person] food. Although they have a hard life, they were healthy, strong, because of their native food. Seal oil, dried fish.”* [Older Native Alaskan, (75) p. 46]

#### Balance—Foundations for a Long Life

Aging well was seen as a combination of the factors reported above and characterized by a holistic approach to living life well and being healthy (15, 66, 70, 72, 73, 75, 77, 80, 83, 84). Several studies referred to the balance in life between physical, spiritual, mental, and emotional realms (15, 77, 80, 83, 84). This often involved working hard, keeping busy, abstaining from drugs, smoking and alcohol, taking positive measures to promote and maintain health, and having an active participation in spiritual and cultural life (15, 66, 73, 75, 77, 83, 84, 89, 90).

“*I live a balanced life without alcohol and drugs. I take care of myself and consciously eat healthy foods regularly, exercise, don't drink or use drugs. Live spiritually.”* [Older Native Alaskan, (77) p. 391]

### Maintaining Connections

Aging well was fostered by the strength of a person maintaining connections to their own identity (66), their family (15, 49, 63–66, 69, 70, 72, 73, 77, 79–83, 85, 89), friends (65, 72), the community (15, 49, 66, 73, 81–83, 89), to their culture (15, 49, 66, 70, 73, 77, 80, 84, 89), spirit (15, 65, 66, 70, 73), and traditional lands (15, 66, 67, 70, 71, 73, 84). These concepts are explored in the sub-themes of: being with my grandchildren keeps me young; people look out for each other; and it's not about age, but about knowledge and wisdom.

#### Being With My Grandchildren Keeps Me Young

A significant theme across studies was the importance of the connection to, and relationships with, kin, which promoted aging well (15, 49, 63–66, 69, 70, 72, 73, 77, 79–83, 85, 89). These relationships often provided the older adult with a sense of purpose in as much as they had a role within the family (66, 77, 81, 82, 85, 89) and were motivated to look after their own health in order to be able to look after their family (66, 73, 89). Participants often reported how looking after grandchildren was rewarding, and a source of joy (63, 65, 66, 73, 85, 89). The wellbeing of the wider family was fundamental to the wellbeing of the older adult (73) and the strong family ties provided opportunities for the older adult to pass on their knowledge and values (15, 66, 83), whilst taking pride in the achievements of the family (65, 83). Families also provided the emotional and physical support that facilitated the older adult staying on traditional lands, and in their own home (83).

“*Being with my grandchildren keeps me young. I love having them around me. We do fun things together, like go to the socials [at an Aboriginal agency] and we smudge [which is a cleansing ritual] at home. I try to help all my grandchildren and support them as much as I can whenever they need it, but I also teach them that they cannot ask for more than they need.”* [Older Aboriginal Canadian, (65) p. 58]

#### People Look Out for Each Other

Integration within community, or social connectedness, was associated with aging well (15, 49, 64–66, 73–78, 80–84, 90). Community involvement was demonstrated with notions of reciprocity, where these relationships provided mutual benefits (71, 73). For older adults, engagement provided an opportunity to socialize, access food, and receive community support with chores such as housework and transport (66, 82–84). In return, communities benefited from older adults sharing their knowledge, wisdom, and experience, as well as assisting the younger generations with guidance and support (15, 66, 77, 80, 82). Socialization through connecting with community gave older adults a sense of belonging, promoted friendships and opportunities to connect with friends, and provided occasions to reminisce and share memories (15, 73, 78, 84). Throughout was the underlying idea of the importance of caring for others (15, 73).

“*…to be able to enjoy life is to be able to live happily with your neighbor. Without that life is not worth it really. I like meeting people and I have a great love for the community that I live in but to make that possible you've got to love the people that are living in that community with you. I like to make myself available for anything, any help that is required regardless. If I can do it, I will do it.”* [Older Maori, (73) p. 207]

Conversely, separation from family, friends and community was shown to have a negative impact on mental health. Increased feelings of loneliness and isolation was perceived by participants to be a major challenge to aging well. This was magnified when close family members left the community or physical disabilities limited access to social events (63, 83, 88, 90).

#### It's Not About age, but About Knowledge and Wisdom

Connection to culture was a significant contributor in perceptions of aging well. This included the ability to pass on traditional values, language, beliefs, wisdom, skills, and knowledge (15, 49, 62, 64–70, 72, 73, 75, 77, 78, 81–83, 85–87, 89, 90, 94). Aging well was promoted though the valuing of older adults, enabling them to fulfill a traditional role, resulting in: a sense of purpose and pride; an identity and meaningful role; an opportunity for continued learning; a means for engaging with family and community; a connection to the natural and spirit worlds; an opportunity for reciprocity with family and community; and allowing the older adults to stay involved in physical activities (15, 49, 65, 66, 69, 72, 73, 75, 77, 81–83, 89, 90). Consequently, through their cultural leadership, the older adults felt needed and respected, had improved emotional wellbeing and optimism, had improved life satisfaction, and gained a sense of accomplishment (15, 49, 66, 73, 78, 81–83, 87, 89, 94). Furthermore, cultural leadership gave a platform to provide advocacy for Indigenous voices, strengthened community cohesiveness, and promoted overall health of the community (49, 66, 73, 89).

“*…I realized I needed to take on a lot of responsibility for the way I acted and the words that I said to people. Now is the time where those my age take all of the teachings we have received and give them back to the community. The community is my extended family. They are all my children; they are all my brothers and sisters; they are the people I love. This is my power. This is what keeps me going, all these people. I really enjoy what I do! I feel great being a part of a community!”* [Older Aboriginal Canadian, (65) p. 60]

### Revealing Resilience, Humor, and a Positive Attitude

Elements of this theme revealed how attitude and an approach to life can impact on the perception of aging well. This includes how older adults used humor in their life (65, 66, 73, 83), demonstrated resilience (15, 65, 66, 73, 78–80, 90), maintained a positive attitude (15, 66, 72–74, 76–79, 83–85, 90), adapted to the changes they face (66, 72–75, 79, 83), and took personal responsibility for their health and wellbeing (15, 66, 73, 77, 83, 94). These concepts are described in the sub-themes of: we're lucky we can laugh at ourselves; you have just to pick yourself up and keep going; and aging well is just being who you are and believing in who you are.

#### We're Lucky We Can Laugh at Ourselves

Humor was used to face the realities of past and present trauma and tragedy and helped participants to maintain a positive attitude (65, 83) and cope with changes (66, 83). In this sense, humor was a form of resilience and the need to seek enjoyment was of importance (63, 73, 83).

“*We're lucky we can laugh at ourselves. During my life, I remember there were so many moments of tragedy and drama. Then I look at it from another viewpoint and think, “How stupid is that?” They are all funny! It's just the whole irony of being alive. You look at it and you think, “That was my life. Good God, I could have done better!” But actually, I could have done worse. Aboriginal people can laugh and don't need to hold a grudge.”* [Older Aboriginal Canadian (65) p. 52]

#### You Have Just to Pick Yourself Up and Keep Going

Resilience and stoicism in the face of adversity was significant across studies (15, 65, 66, 73, 78, 90). Findings highlighted how older adults were aging well, despite experiencing a variety of losses. Participants also demonstrated humility and gratitude for people they had in their lives and the abilities they retained as they aged (65, 66, 80). Having a positive attitude meant facing challenges with courage and not giving up, having a sense of purpose, keeping engaged with life, families, community, and making contributions for the good of the wider community (15, 66, 72, 73, 80, 83, 85). Furthermore, having a positive attitude toward life including demonstrating forgiveness, optimism, belief in oneself, and embracing life, were seen as factors that contributed to aging well (15, 65, 66, 72–74, 76, 78, 79, 84, 90).

“*…you live simply, you live well, you live happily no matter, you are bound to have a few hiccups and some of those hiccups can be dramatic, but you have just to pick yourself up and keep going.”* [Older Māori, (73) p. 217]

Conversely, negative feelings of hopelessness, depression, and worry presented challenges to aging well (74, 77, 87, 90). Three studies referred to older adults' feelings of being a burden on family and community (49, 83, 90).

Having a positive attitude also included the willingness to adjust or adapt to changes in ability or circumstances or changes in roles (66, 72–75, 79, 83). These changes were both personal changes as well as changes within the community such as consequences of assimilation or urbanization. Older adults that were seen to be aging well were able to accept the limitations of old age and make adaptions to accommodate those changes (66, 73, 74, 83, 90).

#### Aging Well Is Just Being Who You Are and Believing in Who You Are

Demonstrating agency and autonomy were seen as factors that promoted aging well with older adults (66, 73, 77, 79, 83, 94). This included gaining and maintaining control over their own life, exercising choice, asserting their needs, managing their own limitations, and staying independent (66, 73, 77, 79, 83). Additionally, taking personal responsibility in managing their health was highlighted. This included having self-awareness and practicing self-care, making good choices in relation to a healthy lifestyle, and taking preventative measures and practicing self-management with regard to chronic disease (15, 66, 73, 77, 94).

“*Aging well in the community is just being who you are and believing in who you are.”* [Older Native Alaskan, (66) p. 63]

### Facing the Challenges

Several factors were documented as challenges to aging well including: access to services or health care (49, 63, 66, 68–70, 73, 83, 85, 88, 90); availability of culturally safe health programs or services (15, 49, 65, 68–70, 73, 74, 85, 86, 88–90); access to resources (63, 68–70, 85, 88, 90); transport (49, 63, 68–70, 73, 83, 85, 88, 90); housing (63, 68, 69, 85); finances (49, 69, 70, 73, 83, 85, 86, 88); and environmental adaptions (63, 68, 85). Impacts of colonization such as: grief and loss (15, 63, 65, 66, 85, 90); loss of language and culture (15, 63, 66, 72, 73, 80, 83–85, 87, 89); and historical trauma (15, 49, 65, 73, 83–86, 89, 90) were also viewed as having an effect on the ability to age well. The elements of this theme are described in the following sub-themes of: services have to be the right fit; comfort of housing and money; and loss.

#### Services Have to Be the Right Fit

Access to mainstream services posed several difficulties for older adults (49, 63, 66, 69, 70, 73, 83, 85, 88, 90). For those living in remote communities, location and availability of services was an issue (63, 68, 69, 88, 90). Lack of transport was frequently cited as being a barrier to service access (15, 49, 63, 68–70, 73, 83, 85, 88, 90). Additionally, older adults were often unaware of the existence services or activities in the local area often as a result of poor information provided to Indigenous communities, or lack of access to computers or Internet to search for or access existing services (63, 68, 70, 85, 88, 90).

“*There are a lot of people who can help, but not knowing where to go or how to go about getting the information is difficult.”* [Older Native Hawaiian, (70) p. 404]

Mainstream services were often reported as expensive and at times were viewed as alienating, promoting further marginalization of Indigenous peoples, and deterring them from accessing care and supports. By contrast, culturally safe programs and services were overwhelming perceived as more appropriate and beneficial. These tended to have a holistic approach, involve community, understand culture and language, and foster trust and respect between providers and older adults (15, 49, 65, 68–70, 73, 74, 85, 86, 88–90).

“*It's important to have an Aboriginal specific program as they feel welcomed here and they see Auntie's and sisters”* [Older Aboriginal Australian, (49) p. 363]

#### Comfort of Housing and Money

Issues with housing were significantly detrimental to the ability to age well. Factors included housing, which was poor quality, expensive, and poorly maintained, as well as a lack of availability resulting in overcrowding (63, 68, 69, 85). Houses were often poorly adapted to health conditions, requiring modifications to enable safety and ongoing independence (63, 68, 85).

“*Main thing is shower is too small and needs to be modified to allow easy access for self and wife. My wife is on walking frame but she will be in a wheelchair sometime in future.”* [Older Aboriginal Australian, (85) p. 40]

Better housing options were those that were affordable, safe, secure, accessible, and supportive of older adults' needs as they aged (68).

Poverty was also a challenge to aging well. Many older adults were living under substantial financial pressure. Lack of adequate finances influenced access to healthy (often more expensive) food, suitable housing, transport, medications, health care, and services (69, 70, 83, 85, 86, 88).

“*I don't get enough money to buy the food I'm supposed to have…I can't buy no fruits and vegetables, they're too expensive.”* [Older First Nations Canadian, (83) p. 72]

#### Loss

The impacts of colonization are deep and wide, and as such, aging needs to be understood through the lens of loss. Loss of family, culture, language, traditions, and land are shared between all Indigenous groups subjected to colonization. Furthermore, social disadvantage, racism, and the ongoing impact of intergenerational and current grief and trauma are all experienced realities (15, 73, 85). Loss of family members and significantly a spouse, meant grieving was an ongoing situation, which affected mental health and wellbeing (63, 65, 66, 85, 90).

“*It takes a lot of coping with and getting over. It's the hardest thing to lose someone in your family. It's very hard”* [Older Aboriginal Australian, (90) p. 7]

Loss of culture and traditional ways has in many cases led to a more sedentary lifestyle (72, 80, 83). Loss of the connections with family and culture was perceived to contribute to a lack of respect from the younger generation (63, 66, 72, 83, 89). Changes in work and social demands mean families spend less time together and the older adults have less opportunity to pass on their knowledge, wisdom, traditions, and language (63, 66, 72, 73, 83).

Historical trauma such as the residential school system, the stolen generations and dispossession of land has impacted on all aspects of Indigenous lives (15, 49, 63, 65, 66, 73, 83–85, 90). The consequences of this trauma are immense and for the aging participants in the reviewed studies included having a lack of a family network to provide support, loss of opportunities, loss of identity, fear and suspicion of governmental services and care—and therefore avoidance of needed supports- and lack of resources to support aging well (49, 65, 73, 83–86, 89, 90).

“*Our life has been interrupted, spiritually and culturally … my people have been hurt … that affected their health. They're lost. It's the loss of their way of life … identity and their culture, their everything. And it's been taken away from them”* [Older Aboriginal Australian, (89) p. 28]

## Discussion

This is the first known systematic search of the literature to scope what aging well means for different Indigenous populations, to compare the described concepts of aging well across these populations, and how the concepts differed to non-Indigenous perceptions of successful aging. Gaps in the literature on aging well for Torres Strait Islander populations was also examined to inform further research.

Concepts of what constitutes aging well are similar between Indigenous and non-Indigenous older adults, with literature reporting physical and mental health, social interactions, and attitude as important for all aging populations (26, 27, 46, 95). The cultural and social determinants of health significantly influence how older adults can age well in their communities whether Indigenous or non-Indigenous (3). However, this review has revealed that from an Indigenous perspective, aging well is a more holistic concept where connections to place, person and culture are interrelated.

Aging well for Indigenous peoples was fundamentally characterized by the component of “engagement with life” proposed by Rowe and Khan, rather than by the components of “lack of illness or disability” or “high cognitive and physical function” that was also included in their model (36). For Indigenous older adults, relationships are essential to aging well (15, 66, 73). Rowe and Khan (36) described active engagement with life as maintenance of interpersonal relationships and productive activity, with interpersonal relations being described as contact with others, exchange of information, emotional support, and direct assistance. Yet for Indigenous older adults, engagement with life was epitomized through the significance of relationality and connectivity, and where interpersonal relationships were more complex. Connections were not solely to maintain contact with others but involved a connection to culture, spirit, place, and whole of community. These relationships provided direction and motivation and the community was viewed as an extension of family. In this respect, Indigenous perspectives took a collective approach to aging well and could not be viewed individually (23). The collective approach to aging well is in contrast to the Western model of successful aging that places the emphasis on the individual (36). These findings align with those described by Pace and Grenier (52), who reviewed perceptions of aging with Indigenous peoples in North America and found relationships with family and community were integral to successful aging.

The importance of connections to traditional land was a significant aspect of aging well across the majority of publications. Indigenous peoples hold a deep connection to their ancestral land and connection to land is central to Indigenous peoples' existence (96). This spiritual connection, created through relationships, is expressed through Indigenous belief and knowledge systems (96). For Indigenous older adults, wellbeing and aging are embedded in their connections to, and relationship with, land (15, 71, 73, 83). Disconnection from their traditional lands compromised health and wellbeing, and impacted on the ability to age well (71, 73, 83).

The importance of generativity- the propensity and willingness to promote the wellbeing of younger generations, contributing to the growth of the next generation—was a further determinant of aging well from an Indigenous North American perspective. The acquisition of material goods or wealth was not considered important for these Elders (65, 66, 78, 81, 82). For Indigenous peoples from across most populations, transmitting their accumulated knowledge, traditional values, and wisdom to the younger generations, and advocating for Indigenous voices, was a fundamental indicator that they had aged well. Aging well helped to establish strong futures for the next generation. Through cultural leadership and as holders of knowledge, older Indigenous adults were shown respect, and were held in high esteem by their communities. This is in contrast to Western society, which, at times, portrays a negative stereotype toward aging adults (97). Within a Western culture, aging adults are commonly depicted as a socioeconomic risk, or a burden on society, as they face frailty and decline with very little to contribute to the overall wealth of the economy (98, 99). These views may well explain why older adults living in Western societies seek to defy aging and prolong youth. In contrast Indigenous peoples report taking joy in aging, as it signifies a time in life where they garner respect and feel valued (100).

Historical and cultural context, disparities, and inequality, are significant in the assessment of aging well (101). The successful aging literature indicates that economic and social privilege facilitates aging well when measured by lack of disease and disability and the maintenance of cognitive and physical function (24, 28, 29, 43). Moreover, in the Rowe and Khan model of successful aging, the success of how well an individual ages is attributed to the individual's choices, effort, and behaviors (36). Yet, lifestyle choices and individual volition are restricted by the accumulative disadvantage across the life course (45). For Indigenous peoples, life course, specifically the impacts of colonization, and the influence of the social determinants of health were ubiquitous across the publications on their perceptions of aging well. The reviewed publications reported that poverty, lack of adequate housing and transport, and ongoing grief and loss posed a challenge to aging well for older Indigenous adults. It is documented that an accumulation of deficits (personal, social, economic, and environmental) predicts ill health and unsatisfactory aging (102). However, positive assets such as resilience, positive attitude, and approach to life, were reported as means to mitigate those negative factors and promote aging well.

This scoping review also aimed to identify gaps in the literature on aging well for specific Indigenous populations, to provide recommendations for further research. The majority of publications were situated in North America (the USA and Canada) and distinguished between their specific Indigenous populations. Whilst six publications focused on Australian Aboriginal and Torres Strait Islander peoples, all included Aboriginal perspectives only with no perspectives specific to the Torres Strait population. Torres Strait Islander peoples are a culturally distinct Indigenous group within Australia, with their own identities formed from different environmental, cultural, and historical circumstances (51). Promotion of local programs for aging well requires a context-specific approach based on the concerns that local older adults find essential to their health (103). Understanding of local needs could decrease barriers to culturally appropriate health care (103), whilst supporting a holistic view of functioning and healthy aging (3). Therefore, research that explores concepts of aging well specifically for Torres Strait Islander peoples is required, and will address a gap in the existing literature.

## Implications of Findings

Access to culturally appropriate health services and support programs remain a challenge for older Indigenous adults. A range of barriers was reported including locality, transport, cost, and cultural appropriateness. To successfully engage with older Indigenous adults and achieve program objectives, culturally safe approaches to care are critical (15, 90, 104, 105). Existing health care programs often neglect the cultural safety needs of Indigenous peoples (90) and do not consider the views of the consumer using the service (106). This can result in poor access to, and perceived non-compliance with, services (90). The findings of the review suggest any aging well programs or support services should take a culturally safe, holistic, multifaceted, and whole-of-community approach. Models of aging well also need to account for the complex health conditions that arise from the inequalities across the life course and the social determinants of health that influence aging (52). Empowering individuals to recognize and build on their strengths (resilience, attitude, and approach to life), may help promote their health status and aging trajectory (102). A strengths-based approach to aging well values the skills, knowledge, and relationships to both older adults and their communities.

## Limitations

This scoping review had some limitations. Only articles published in English were included, thus potentially excluding studies of Indigenous peoples from non-English speaking nations such as inclusion of Indigenous peoples from Africa, Asia, the Pacific Islands, and Europe. The databases used in the scoping review were chosen due to their wide-spread use within the Australian context. Indigenous-focused databases or websites specific to countries other than Australia, or in languages other than English, may have additional publications of relevance not identified. The majority of studies (excluding those from Ecuador and Chile) were from English-speaking nations that had a common history of colonization by Britain, although this was not an inclusion criteria. As such, this may reduce the generalisability of the findings. Further research is needed to effectively explore aging for Indigenous peoples of other continents, different languages, and those without a history of colonisiation. A further limitation arose due to differences in original research methodologies of the included studies. Differences in the importance of domains of aging well may have been influenced by specific questions asked of participants across studies. Some studies had predetermined domains of aging identified and specific topic areas, whilst other studies had more open-ended questioning formats. The application of the quality appraisal tool had its own limitations. A Western approach to appraising publications may not be suitable for all types of data—for this reason the autobiographical account was not rated but deemed valuable in adding to the understanding of aging well from an Indigenous perspective. Finally, findings synthesized from the reviewed publications were of a secondary source. As such, these findings were dependent on the rigor and trustworthiness of the primary authors in interpreting their results so that they accurately represented the voices of the Indigenous participants.

## Conclusion

This scoping review presents concepts of what aging well means for different Indigenous peoples, providing an insight into how these perspectives differ from a non-Indigenous aging well model. Aging well for Indigenous peoples is a holistic concept where connections to culture, land and the wider community are integral. The literature reviewed highlighted the challenges common to Indigenous populations to achieve good health and wellbeing as they age. Gaps in perspectives from specific Indigenous populations, such as Torres Strait Islander peoples in Australia, has been identified, which highlights the call for locally conducted research into the specific needs of this population. Opportunities exist for health service and social support providers to develop strengths-based, culturally safe programs that better align health and social care systems to integrate services that support a holistic and positive view of aging well.

## Data Availability Statement

Publicly available datasets were analyzed in this study and the details of which are included in the article. Further inquiries can be directed to the corresponding author/s.

## Author Contributions

RQ, SR, MR-M, and SL contributed to the conception and design of the study. RQ completed the literature search, led the analysis with input from SR and MR-M, and wrote the manuscript. RQ and SR reviewed all articles. MR-M and SL provided consensus where required. All authors contributed to manuscript revision, read, and approved the submitted version.

## Funding

Open Access publication fees paid by College of Medicine and Dentistry, James Cook University.

## Conflict of Interest

The authors declare that the research was conducted in the absence of any commercial or financial relationships that could be construed as a potential conflict of interest.

## Publisher's Note

All claims expressed in this article are solely those of the authors and do not necessarily represent those of their affiliated organizations, or those of the publisher, the editors and the reviewers. Any product that may be evaluated in this article, or claim that may be made by its manufacturer, is not guaranteed or endorsed by the publisher.
